# Mycobiota and Mycotoxins in Traditional Medicinal Seeds from China

**DOI:** 10.3390/toxins7103858

**Published:** 2015-09-24

**Authors:** Amanda Juan Chen, Xiaolin Jiao, Yongjian Hu, Xiaohong Lu, Weiwei Gao

**Affiliations:** Institute of Medicinal Plant Development, Chinese Academy of Medical Sciences and Peking Union Medical College, Beijing 100193, China; E-Mails: amanda_j_chen@163.com (A.J.C.); jiao_1110@163.com (X.J.); hu713th@163.com (Y.H.); luchauluchau@gmail.com (X.L.)

**Keywords:** medicinal seeds, mycobiota, UPLC-MS/MS, aflatoxins, ochratoxin A, mycotoxigenic fungi

## Abstract

The multi-mycotoxin occurrence for internal and superficial fungi contamination were comprehensively assessed in medicinal seeds used as food or beverage. Based on a polyphasic approach using morphological characters, β-tubulin and ITS gene blast, a total of 27 species belonging to 12 genera were identified from surface-sterilized seeds. *Chaetomium globosporum* was most predominant (23%), followed by *Microascus trigonosporus* (12%) and *Alternaria alternata* (9%). With respect to superficial mycobiota, thirty-four species belonging to 17 genera were detected. *Aspergillus niger* and *Penicillium polonicum* were predominant (12% and 15%, respectively). Medicinal seed samples and potential toxigenic fungi were tested for ochratoxin A (OTA) and aflatoxins (AFB1, AFB2, AFG1, AFG2) using UPLC-MS/MS. Platycladi seeds were contaminated with AFB1 (52.0 µg/kg) and tangerine seed was contaminated with OTA (92.3 µg/kg). Subsequent analysis indicated that one *A. flavus* strain isolated from platycladi seed was able to synthesize AFB1 (102.0 µg/kg) and AFB2 (15.3 µg/kg). Two *P. polonicum* strains isolated from tangerine and lychee seeds were able to synthesize OTA (4.1 µg/kg and 14.8 µg/kg, respectively). These results identify potential sources of OTA and aflatoxins in medicinal seeds and allude to the need to establish permitted limits for these mycotoxins in these seeds that are commonly consumed by humans.

## 1. Introduction

According to the World Health Organization, 80% of people living in developing countries rely on traditional herbal medicines [1]. Herbal and other alternative treatments are also popular in the developed world, where they are used by about half of Australians and one third of Americans [2,3]. China is one of the largest producers of traditional herbal medicines (THM) in the world. According to 2011 Chinese customs statistics, 2.3 billion dollars of THM were exported to Japan, the United States and European Union, among other countries [4]. In THM, parts of botanicals such as roots, leaves, flowers, seeds, *etc.*, are separately used to acquire the best pharmaceutical effect. So far, more than 60 medicinal seeds have been recorded in the pharmacopoeia of China [5]. Many of these are not only used medically; some medicinal seeds such as coix seed, lotus seed and cassia seed are also considered dietary herbs, which are widely used in foods and health drinks [6,7].

Medicinal seeds are commonly infected in the field (pre-harvest) or during storage (post-harvest) with various molds which may result in the production of mycotoxins. Currently, more than 400 mycotoxins have been identified, but the most significant classes of mycotoxins [8], with respect to their toxic or carcinogenic properties, include aflatoxins (AFs) [9] and ochratoxin A (OTA) [10]. AFs are hepatotoxic, teratogenic, mutagenic and carcinogenic mycotoxins produced by members of *Aspergillus* section *Flavi*, in particular *A. flavus* and *A. parasiticus* [11]. The most potent of the four naturally occurring AFs (B1, B2, G1 and G2) is aflatoxin B1 (AFB1), which is listed as a group I carcinogen by the International Agency for Research on Cancer [9] because of its demonstrated carcinogenicity to humans.

Apart from AFs, OTA is one of the most important mycotoxins and the most toxic member of the ochratoxin group. OTA exhibits teratogenic, embryotoxic, genotoxic, neurotoxic, immunosuppressive, carcinogenic [12], and nephrotoxic effects [13,14]. The most important ochratoxigenic *Aspergillus* spp. are members of *Aspergillus* section *Nigri* and section *Circumdati* [15,16,17]. *Penicillium nordicum* and *P. verrucosum* are the primary OTA producers in the *Penicillium* genera [18,19]. In addition, *Penicillium* spp. including *P. chrysogenum*, *P. glycyrrhizacola* and *P. polonicum* have been described as a primary source of OTA contamination in liquorice roots [20,21].

Due to AFs’ toxicological effects, the maximum permitted levels in several medicinal herbs have been set in China, to 5 µg/kg for AFB1 and 10 µg/kg for the sum of AFB1, AFB2, AFG1 and AFG2 [5]. With respect to OTA, the maximum permitted level was set in liquorice root to 20 µg/kg by the European Commission (EC) Regulation No 105/2010 [22] and amending Regulation 1881/2006 [23]. The problems of AFB1 contamination have been well documented in several medicinal seeds including platycladi seed and coix seed [24,25,26]. However, little research has been conducted to describe the occurrence of OTA and mycotoxigenic fungi. The purpose of the present study is: (1) to determine the simultaneous occurrence of aflatoxins (AFB1, B2, G1, G2) and OTA in twelve commonly used medicinal seeds; and (2) to determine the mycobiota associated with these medicinal seeds and their potential ability to produce mycotoxins.

## 2. Results and Discussion

### 2.1. Water Activity 

**Figure 1:**
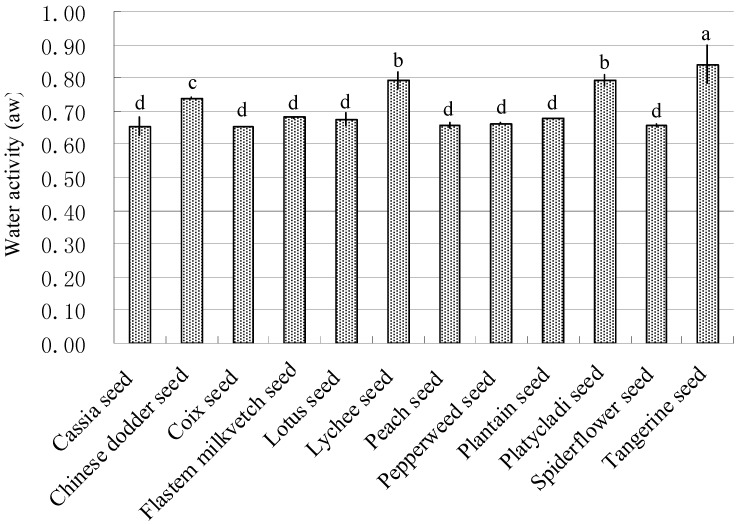
Water activity of tested medicinal seeds. The data are means ± SD (*n* = 3), and data were analyzed using an LSD test. The same letters above each of the bars indicate no significant difference in mean values of water activity of seed samples (*p* ≥ 0.05), while different letters indicate significant difference (*p* < 0.05).

The water activity (a_w_) of the studied medicinal seeds ranged from 0.65 ± 0.00 to 0.84 ± 0.06 (Figure 1). The highest water activity was found in tangerine seeds (0.84 ± 0.06 a_w_), followed by lychee seeds (0.79 ± 0.02 a_w_), platycladi seeds (0.79 ± 0.02 a_w_), and Chinese dodder seeds (0.74 ± 0.00 a_w_).

### 2.2. Internal Fungal Incidence

Different infection frequencies that ranged from 2% to 100% were observed, except that no internal fungi were acquired from pepperweed and flastem milkvetch seed samples (Table 1). A total of 352 fungal isolates belonging to 12 genera and 27 species were detected from infected seeds. *Chaetomium globosporum* was the most commonly identified fungi, which could be detected in 4 out of 10 medicinal seed samples. Isolates related to *C. globosporum* represented 23% of the total internal fungal counts, whereas isolates related to *Microascus trigonosporus* and *Alternaria alternata* accounted for 12% and 9% of total internal fungal counts, respectively.

**Table 1:** Internal fungal incidence (CFU/100 seed) in studied medicinal seeds ^a^.

Fungal Species	Tangerine Seed	Lychee Seed	Platycladi Seed	Spiderflower Seed	Peach Seed	Coix Seed	Plantain Seed	Lotus Seed	Cassia Seed	Chinese Dodder Seed	Sum	Frequency %
*Acremonium* sp.		9									9	2.56
*Alternaria alternata*				28					2		30	8.52
*Aspergillus flavus*			25							1	26	7.39
*Aspergillus fumigatus*		15	14								29	8.24
*Aspergillus nidulans*		20									20	5.68
*Aspergillus niger*			27								27	7.67
*Aspergillus proliferans*		2									2	0.57
*Aspergillus steynii*	2										2	0.57
*Aspergillus tubingensis*	2										2	0.57
*Aspergillus versicolor*	2										2	0.57
*Botryophaeria* sp.			1								1	0.28
*Chaetomium globosporum*	39	16			22		4				81	23.01
*Cochliobolus sativus*						2					2	0.57
*Eurotium amstelodami*		19									19	5.4
*Eurotium chevalieri*		3									3	0.85
*Eurotium repens*	5		1								6	1.7
*Eurotium rubrum*		16									16	4.55
*Fusarium incarnatum*						2					2	0.57
*Microascus cirrosus*	5										5	1.42
*Paecilomyces variotii*		2									2	0.57
*Penicillium aurantiogriseum*								2			2	0.57
*Penicillium bilaiae*	6										6	1.7
*Penicillium polonicum*	7			2						1	10	2.84
*Penicillium solitum*	3										3	0.85
*Penicillium spinulosum*	2										2	0.57
*Phoma* sp.						2					2	0.57
*Microascus trigonosporus*	41										41	11.65
Infected seeds No. /100 seeds	100	92	59	30	22	6	4	2	2	2		
Seeds infected with more than two fungal species	20	9	10	0	0	0	0	0	0	0		
Co-infected fungal species	*Chaetomiun globosporum *+ *Penicillium* spp.	*Eurotium* spp. + *Aspergillus* spp./ *Eurotium* spp. + *Chaetomium globosporum*	*Aspergillus niger *+ *Aspergillus flavus*									

^a^ none internal fungi were acquired from pepperweed seed and flastem milkvetch seed samples.

Tangerine, lychee, and platycladi seeds exhibited the highest fungal infection rates, which may be related to their elevated water activity. Among them, 100% of tangerine seeds was found to be infected by fungi and 20% were co-infected with *C. globosporum* and *Penicillium* spp. (*P. bilaiae*, *P. polonicum*). Ninety-two percent of lychee seeds were found to be infected by fungi and 9% were co-infected with *Eurotium* spp. (*E. amstelodami*, *E. chevalieri*, *E. rubrum*) and *Aspergillus* spp. (*A. fumigatus*, *A. nidulans*) or with *Eurotium* spp. (*E. amstelodami*, *E. chevalieri*, *E. rubrum*) and *C. globosporum*. Fifty-nine percent of platycladi seeds were found to be infected with fungi and 10% were co-infected with *A. niger* and *A. flavus*.

Plant seeds are rich in starch, protein, and fat and are thus highly susceptible to fungal contamination and growth during harvesting and storage. A number of survey and monitoring programs have been carried out in several countries that have attempted to define a general pattern of the contaminated mycobiota in association with edible seeds. These included maize, cocoa beans, peanut, and other cereals. Mycobiota associated with these seeds included *Aspergillus* spp., *Penicillium* spp., *Fusarium* spp., and *Alternaria* spp. [27,28,29,30,31,32]. By contrast, little research has been focused on characterizing the mycobiota that associate with medicinal seeds. Kong *et al.* [25] analyzed the mycobiota associated with 4 medicinal seeds including coix, lotus, bitter apricot, and ginkgo seeds. Their results indicated that coix seed was most contaminated (contamination confirmed in 5%–9% of total seeds) followed by lotus seed (contamination confirmed in 7% of total seeds), ginkgo seed (contamination confirmed in 6% of total seeds) and bitter apricot seed (contamination confirmed in 4% of total seeds). The contaminating mycobiota included *A. flavus* section, *A. niger* section and *A. ochraceus* section. According to our findings, 4% of coix seeds were infected with *F. incarnatum* and *Cochliobolus sativus* and 2% of lotus seeds were infected with *P. aurantiogriseum*. Different mycobiota were commonly observed in other edible seeds too, such as in maize, *A. niger* and *A. flavus* were predominant maize mycobiota when cultivated in hot climatic conditions, while *Fusarium* spp. was more prevalent when maize was cultivated in a warm climate [27,29]. Another alternative explanation is that field fungi including *Fusarium*, *Alternaria,* and *Cochliobolus* infect seeds at an early stage during plant growth in the field, whereas *Penicillium* and *Aspergillus* tend to infect seeds during storage, in particular when storage conditions are poor [33,34,35,36].

Compared with other medicinal seeds, tangerine and lychee seeds are coated with juicy, sugary pulp or aril, which is difficult to completely remove during the processing procedure. This substrate may increase the susceptibility of these two seeds to high fungal contamination. With respect to platycladi seeds, the high fatty oil content (12%–51%) may increase the susceptibility of these seeds to fungal infection [37]. Numerous studies have documented the effect of lipids on the growth of the fungi *A. flavus* and *A. parasiticus*. Mellon *et al.* [38] determined that *A. flavus* growth was more supported on media with triglycerides as a sole carbon source than observed on raffinose reference medium. In addition, AF production levels were found to be 800-fold higher when *A. flavus* was grown with lipid-rich substrates compared to growth with the same substrate without lipid.

Co-infections were found between *C. globosporum* and *Penicillium* spp., *Eurotium* spp. and *Aspergillus* spp., *Eurotium* spp. and *C. globosporum,* and *A. niger* and *A. flavus*. Co-infections with *A. niger* and *A. flavus* have been reported in several studies [29,31,39], and are likely attributable to the fact that sections *Flavi* and *Nigri* share common habitats and physiological characteristics. Thus, conditions that favor the growth of one of these fungi probably favor the growth of the other [39,40,41]. Alternatively, such results may reflect facilitative interactions between these populations, whereby growth of one strain creates conditions (e.g., nutrients) conducive to the growth of the other strain. The positive correlation observed between co-infection of *C. globosporum* and other fungi in medicinal seeds may be due to a similar phenomenon, since this fungus is widespread and could produce high amounts of cellulase complex [42]. The cellulase produced might benefit other fungi during infection of medicinal seeds. Recently *C. globosporum* was detected in cumin (*Cuminum cyminum*) and aniseed (*Pimpinella anisum*) too [43].

### 2.3. Incidence of Superficial Fungi on Seed Surfaces

Total fungal counts ranged from 6.5 × 10^1^ to 8.1 × 10^4^ CFU/g seed. Among the twelve kinds of medicinal seeds tested, flastem milkvetch, coix, lychee, lotus, and Chinese dodder seeds exhibited lower levels of fungal contamination, which ranged from 6.5 × 10^1^ to 4.5 × 10^2^ CFU/g. The other seven medicinal seeds were highly contaminated, with fungal counts ranging from 6.75 × 10^3^ to 8.1 × 10^4^ CFU/g, (Table 2). The superficial fungal counts have little to do with the seed size.

A total of 34 species of fungi belonging to 17 genera were detected among these CFUs (Table 2). *Aspergillus*, *Penicillium*, *Mucor*, and *Fusarium* were the four most predominant genera detected in association with all medicinal seeds (exception being spiderflower seed) which represented about 41%, 29%, 6% and 5% of the total CFUs, respectively. The most common genus identified was *Aspergillus* and this was represented by 9 species, among which *A. niger* was most predominant (about 12% of total), followed by *A. versicolor* (about 7% of total), *A. tubingensis* (about 7% of total), and *A. fumigatus* (about 5% of total). The second common genus, *Penicillium*, was represented by 9 species, among which *P. polonicum* was most predominant. *P. polonicum* was isolated from 10 of the 12 medicinal seed samples and represented about 15% of the total CFUs, followed by *P. citrinum* (about 5% of total) and *P. aurantiogriseum* (about 5% of total).

Comparison between different medicinal seeds revealed *A. niger* as the most predominant species in association with platycladi seed and Chinese dodder seed, representing 72% and 28% of the total CFUs, respectively. *A. fumigatus*, *A. sydowii*, *A. tubingensis*, *A. versicolor*, *F. incarnatum*, *M. racemosus*, *Paecilomyces variotii*, *P. aurantiogriseum*, *P. citrinum* and *P. polonicum* were predominant in plantain, pepperweed, lotus, cassia, spiderflower, flastem milkvetch, coix, peach, tangerine and lychee seeds, respectively.

In this study, large numbers of superficial fungi were observed and low similarity was found between superficial and internal mycobiota. Bayman *et al.* [39] also reported that the abundance of *Aspergillus* spp. and *Penicillium* spp. of surface-sterilized pistachio nuts were significantly lower than that of unsterilized nuts.

*F. incarnatum* and *A. alternate* were the two most predominant species associated with spiderflower seed, where they represented about 51% and 33% of the total CFUs, respectively. Surprisingly, *Aspergillus* spp. and *Penicillium* spp. were not detected. The reason for this observation may be that spiderflower seed is not a suitable substrate for *Aspergillus* spp. and *Penicillium* spp. An alternative explanation is that competitive interactions exist between *Fusarium* spp. and *Aspergillus* spp./*Penicillium* spp. In support of the latter hypothesis, several studies have previously reported a negative correlation between the distribution of *Fusarium* spp. and *Penicillium* spp. in corn grains [44,45].

**Table 2:** Superficial fungal incidence (%) in studied medicinal seeds. The percent of CFUs associated with a given genus are given for each seed.

Fungal Species	Tangerine Seed	Lychee Seed	Platycladi Seed	Spiderflower Seed	Peach Seed	Coix Seed	Plantain Seed	Lotus Seed	Cassia Seed	Chinese Dodder Seed	Flastem Milkvetch Seed	Pepperweed Seed	Relative Frequency %
*Absedia* sp.							8.00						0.67
*Acremonium* sp.											3.03		0.25
*Alternaria alternata*				33.33								5.88	3.27
*Aspergillus candidus*						4.11							0.34
*Aspergillus flavus*	12.12		23.03		2.67						3.03		3.40
*Aspergillus fumigatus*		9.84			5.33	2.74	40.00		3.70				5.13
*Aspergillus nidulans*					1.33					5.56			0.57
*Aspergillus niger*			72.47		22.67		16.00			28.00	9.09		12.35
*Aspergillus ochraceus*		31.15						8.00					3.26
*Aspergillus sydowii*								4.00			12.12	23.53	3.30
*Aspergillus tubingensis*								56.00			12.12	5.88	6.17
*Aspergillus versicolor*							4.00	4.00	66.67			5.88	6.71
*Bispora* sp.												5.88	0.49
*Chaetomium globosporum*										5.56	3.03	5.88	1.21
*Cladosporium cladosporioides*				13.89				12.00			6.06		2.66
*Eurotium repens*						16.44							1.37
*Fusarium incarnatum*				50.93									4.24
*Fusarium solani*												11.76	0.98
*Mucor racemosus*					1.33		12.00	4.00	3.70	26.00	27.27		6.19
*Paecilomyces variotii*						64.38							5.37
*Penicillium aurantiogriseum*					65.33								5.44
*Penicillium chrysogenum*		3.28									3.03		0.53
*Penicillium citrinum*	48.48		4.49					4.00			3.03	5.88	5.49
*Penicillium commune*						1.37			3.70				0.42
*Penicillium implicatum*										5.56			0.46
*Penicillium oxalicum*	1.21			1.54							3.03		0.48
*Penicillium polonicum*	38.18	55.74			1.33	5.48	20.00	4.00	18.52	11.11	15.15	11.76	15.11
*Penicillium variabile*								4.00					0.33
*Penicillium viridicatum*									3.70				0.31
*Phialophora* sp.						4.11							0.34
*Phoma* sp.												5.88	0.49
*Scopulariopsis* sp.												11.76	0.98
*Trichoderma koningii*										11.11			0.93
*Vlocladium* sp.						1.37				5.56			0.58
Total fungal counts (CFU/g)	4.13 × 10^4^	1.53 × 10^2^	4.25 × 10^4^	8.1 × 10^4^	1.88 × 10^4^	1.83 × 10^2^	6.25 × 10^4^	6.5 × 10^1^	6.75 × 10^3^	4.5 × 10^2^	8.25 × 10^1^	5.75 × 10^4^	

### 2.4. Occurrence of Mycotoxins (Aflatoxins and OTA) in Medicinal Seeds and Fungal Cultures 

**Figure 2:**
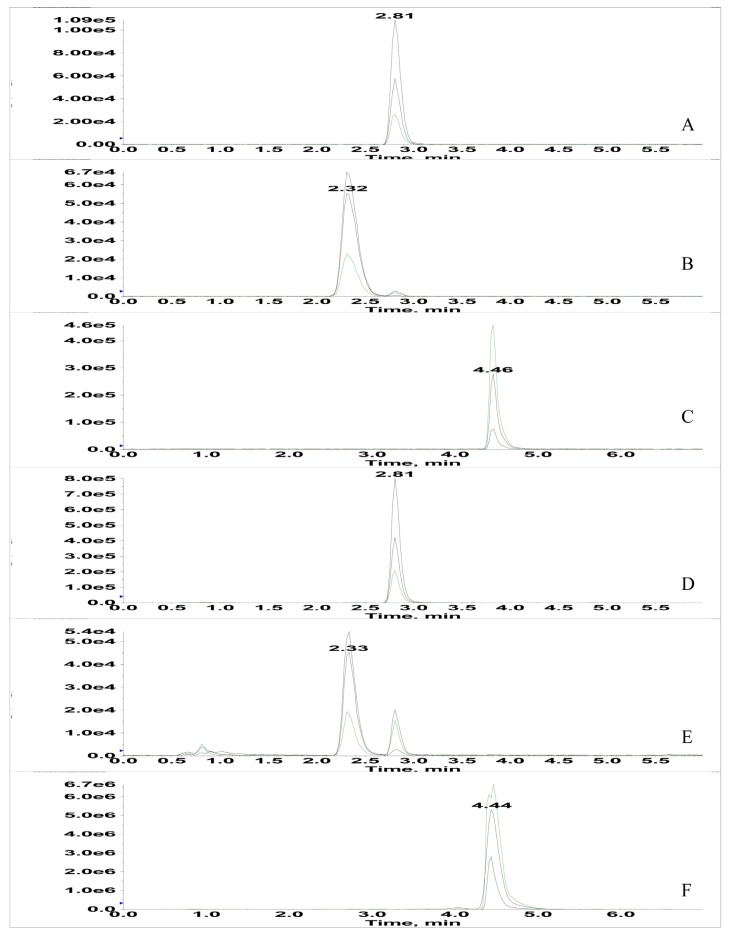
UPLC-MS/MS chromatography with MRM modes for (**A**) AFB1 standard; (**B**) AFB2 standard; (**C**) OTA standard; (**D**) AFB1 positive strain; (**E**) AFB2 positive strain; (**F**) OTA positive seed sample. Three precursor-to-product ion transitions were simultaneously monitored at *m/z* 313.0–285.1, *m/z* 313.0–241.3, *m/z* 313.0–185.1 for AFB1; *m/z* 315.0–259.0, *m/z* 315.0–287.0, *m/z* 315.0–243.0 for AFB2; 329.0–243.0, 329.0–311.0, 329.0–283.0 for AFG1; *m/z* 331.0–235.1, *m/z* 331.0–257.0, *m/z* 331.0–189.0 for AFG2; and *m/z* 404.0–358.1, *m/z* 404.0–341.1, *m/z* 404.0–239.2 for OTA.

Co-occurrence of aflatoxins and OTA were determined by an optimized UPLC-MS/MS method (Figure 2). The frequency of aflatoxins and OTA was low in analyzed medicinal seed samples. Aflatoxin B1 was only detected in platycladi seeds, and OTA was only detected in tangerine seeds. However, the contamination levels were very high, 52.0 µg/kg for AFB1 in platycladi seeds and 92.3 µg/kg for OTA in tangerine seeds. The high AFB1 levels (5.61–27.8 µg/kg) in platycladi seeds have been reported in several studies [24,26]. Accordingly, the maximum permitted levels of aflatoxins in this seed have been established in China (5 µg/kg for AFB1 and 10 µg/kg for the sum of AFB1, AFG1, AFB2, and AFG2) [5]. In contrast, OTA occurrence in medicinal seeds has rarely been studied, and this is the first report concerning high OTA levels in tangerine seeds. Considering the vulnerability of several traditional Chinese medicines to OTA, we suggest that a maximum permitted level for this mycotoxin is urgently needed.

Forty strains of 6 potentially toxigenic species including *A. flavus*, *A. niger*, *A. ochraceus*, *A. tubingensis*, *P. chrysogenum* and *P. polonicum* were screened for aflatoxin and OTA production. AFB1 and AFB2 were detected in one *A. flavus* strain (strain number: Platycladi-3-3) isolated from platycladi seeds at levels of 102.0 µg/kg and 15.3 µg/kg, respectively, suggesting this fungus as the source of AFB1 contamination in platycladi seeds. The sequence of the beta-tubulin gene from this strain was deposited in GenBank with accession number KT737451 (Table S1). The other four *A. flavus* strains isolated from flastem milkvetch, tangerine, peach and Chinese dodder seeds were not found to produce AFs. Several studied reported that the highly variable aflatoxigenic profile of *A. flavus* populations seems to depend as much on the geographic region where the organism was isolated as on the growth substrate. For example, *A. flavus* isolates from peanuts seem to be predominantly aflatoxigenic (70%–100% of all isolates) which is substantially higher than in other crops, independent of the geographic region where the crops were grown [35,46,47]. In this study, platycladi seeds seem to be a more suitable substrate for aflatoxigenic strains when compared to other medicinal seeds. OTA could be detected in one *P. polonicum* strain isolated from tangerine seeds (strain number: Tangerine-3-3) and one *P. polonicum* strain isolated from lychee seeds (strain number: Lychee-2-2) at levels of 4.1 µg/kg and 14.8 µg/kg, respectively. The sequences of the beta-tubulin gene from the two strains were deposited in GenBank with accession numbers KT737453 (Table S2) and KT737452 (Table S3). This fungal genus has been implicated as a primary OTA contributor in liquorice root [20,21]. It seems that this fungus is also an important OTA contributor in medicinal seeds because of its wide distribution and toxigenic features. However, since we only detect several *P. polonicum* isolates in this experiment, the ability of this species to produce OTA still needs to be confirmed in further studies. AFs and OTA were not detected in the other 4 species.

## 3. Experimental Section

### 3.1. Sample Collection

**Figure 3:**
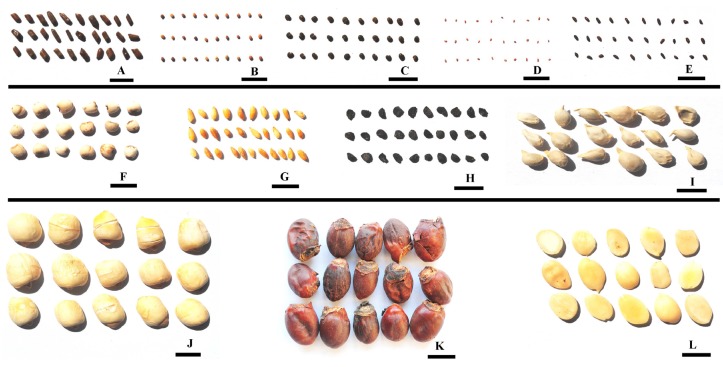
Seed samples. (**A**) Cassia seed; (**B**) Chinese dodder seed; (**C**) Flastem milkvetch seed; (**D**) Pepperweed seed; (**E**) Plantain seed; (**F**) Coix seed; (**G**) Platycladi seed; (**H**) Spiderflower seed; (**I**) Tangerine seed; (**J**) Lotus seed; (**K**) Lychee seed; (**L**) Peach seed. Bar = 10 mm.

Twelve kinds of medicinal seeds (Figure 3) were chosen on the basis of commercial availability and popularity, and were collected randomly from Anguo herbal market, Hebei province, China. The Anguo herbal marker is one of the four largest herbal markets in China. All samples were stored for 1 year before collection. For each kind of medicinal seed, about 500 g of sample were collected from each of 5 different herbal stores and put into sterile paper bags to obtain 2500 g of total weight. All samples were identified by Prof. Bengang Zhang, Institute of Medicinal Plant Development, Chinese Academy of Medical Sciences & Peking Union Medical College, Beijing, China. The names, original plant, and producing regions of twelve medicinal seeds are presented in Table 3. Water activity of all medicinal seeds was measured in triplicate with a LabMaster-aw water activity measurement instrument (Novasina AG, Lachen, Switzerland) at 25 °C. Seeds that were large (including tangerine seeds, lychee seeds and lotus seeds) were cut into small pieces to facilitate internal measurements.

**Table 3:** Names, original plant and producing regions of studied medicinal seeds.

No.	Name	Original Plant	Producing Regions
1	Cassia seed	*Cassia obtusifolia* L.	Hebei province
2	Chinese dodder seed	*Cuscuta chinensis* Lam.	Inner Mongolia autonomous region
3	Coix seed	*Coix lachryma-jobi* L. var. ma-yuen (Roman.) Stpf	Guizhou province
4	Flastem milkvetch seed	*Astragalus complanatus* R. Br.	Gansu province
5	Lotus seed	*Nelumbo nucifera* Gaertn.	Shandong province
6	Lychee seed	*Litchi chinensis* Sonn.	Guangxi autonomous region
7	Peach seed	*Prunus persica* (L.) Batsch	Shandong province
8	Pepperweed seed	*Lepidium apetalum* Willd.	Hebei province
9	Plantain seed	*Plantago asiatica* L.	Liaoning province
10	Platycladi seed	*Platycladus orientalis* (L.) Franco	Shandong province
11	Spiderflower seed	*Cleome gynandra* L.	Hebei province
12	Tangerine seed	*Citrus reticulata* Blanco	Guangdong province

### 3.2. Isolation of Fungi

Two different isolation methods were used to analyze the internal and superficial contaminating fungi. For internal fungal isolation, 100 of each medicinal seeds were surface sterilized using 75% ethanol for 1 min, followed by treatment with NaClO (3% available chlorine) for 3 min, and finally a second 75% ethanol wash for 1 min [48]. The seeds were then dried on sterilized paper and 100 of each sample were aseptically plated in malt extract agar (MEA, 20 g of malt extract, 1 g of peptone, 20 g of dextrose, 15 g of agar, and brought up to 1 L with distilled water) dishes containing 50 ppm tetracycline and 100 ppm streptomycin. All plates were incubated at 25 °C and were examined for growth daily. For superficial fungal isolation, 10 g of each seed sample was added to 90 mL sterile water and mixed. This mixture was then shaken on a rotary shaker for approximately 30 min and subjected to a series of ten-fold serial dilution to a final dilution of 10^−3^. Aliquots consisting of 1 mL of each dilution were spread (in triplicate) on MEA containing 50 ppm tetracycline and 100 ppm streptomycin. One of the three sets of dilutions that averaged between 10 and 60 colonies per plate was selected for enumeration. The results of triplicate plating are expressed as the average CFU/g.

### 3.3. Identification of Fungi 

Followed by preliminary morphological identification, every fungal colony was transferred and re-streaked onto MEA. With respect to *Penicillium* spp. and *Aspergillus* spp., colonies were streaked on Czapek Yeast Autolysate Agar (CYA, 1 g of K_2_HPO_4_, 10 mL of Czapek concetrate, 5 g of yeast extract, 30 g of sucrose, 15 g of agar, and brought up to 1 L with distilled water) and Yeast Extract Sucrose Agar (YES, 20 g of yeast extract, 150 g of sucrose, 0.5 g of MgSO_4_·7H_2_O, 20 g of agar, and brought up to 1 L with distilled water) and were incubated at 25 °C for 7 days. Colony colour was assessed according to The Methuen Handbook of Colour by Kornerup and Wanscher [49]. Other macroscopical and microscopic morphological observations (e.g. colony texture, conidiophore and conidia characteristics) were made according to previously published methods [50,51,52,53]. To verify the results of morphological characterization and identification of fungi, DNA of each strain was extracted using a commercial DNA extraction kit according to the instructions (Biomed Co., Ltd., Beijing, China). The β-tubulin gene was PCR amplified and sequenced [54]. With respect to fungal species other than *Penicillium* spp. and *Aspergillus* spp., the ITS gene was amplified and sequenced [55]. Obtained sequences were manually trimmed at the N and C terminals to delete low quality ends, and Basic Local Alignment Search Tool (BLAST) was used to identify the closest affiliated sequence in the GenBank/NCBI dataset.

### 3.4. Mycotoxin Production by Fungal Isolates

Fungal strains were cultured and extracted according to Chen *et al.* [21] with several slight modifications. About 5 g dry fungal culture was blended with 100 mL methanol-water (80/20) and extracted using ultrasonography for 45 min. The mixture was then filtered using quantitative filter paper. The filtrate was collected with an evaporating dish and was dried for 24 h at 60 °C. The dried residue was then dissolved with 10 mL methanol/water (80/20) and was purified and concentrated by a solid phase extraction (SPE) column (NERCB-SPE, 100 mg/3 mL, National Engineering Research Center for Biotechnology, Beijing, China). Each sample was filtrated through a 0.22 μm PTFE filter before UPLC-MS/MS analysis. UPLC-MS/MS analysis was performed with a LC-20AD_XR_ Prominence Liquid chromatograph (Shmadzu Corp., Kyoto, Japan) interfaced to a 5500 QTRAP MS system (AB Sciex Pte. Ltd., Framingham, MA, USA). The sample was separated using a ZORBAX Eclipse Plus C18 column (2.1 × 50 mm, 1.8-Micron, Agilent Technologies Co., Ltd., Santa Clara, CA, USA). The mobile phase consisted of (A) acetonitrile containing 0.1% formic acid and (B) water containing 0.1% formic acid, The gradient procedure was as follows: 0.01–4.00 min, 70%–20% B; 4.00–5.00 min 20%–70% B; 5.00–6.00 min 70% B. The mass spectrometer was operated in the positive mode with Multiple Reaction Monitoring (MRM) at unit mass resolution. Three precursor-to-product ion transitions were simultaneously monitored at *m/z* 313.0–285.1, *m/z* 313.0–241.3, *m/z* 313.0–185.1 for AFB1; *m/z* 315.0–259.0, *m/z* 315.0–287.0, *m/z* 315.0–243.0 for AFB2; 329.0–243.0, 329.0–311.0, 329.0–283.0 for AFG1; *m/z* 331.0–235.1, m/z 331.0–257.0, *m/z* 331.0–189.0 for AFG2; and *m/z* 404.0–358.1, *m/z* 404.0–341.1, *m/z* 404.0–239.2 for OTA. The MRM model was used for quantification of peaks. The limits of detection (LOD) and the limits of quantification (LOQ) were less than 0.05 and 0.1 µg/kg, respectively.

### 3.5. Analysis of Medicinal Seed Samples to Detect Natural Occurrence of AFs (AFB1, AFB2, AFG1, AFG2) and OTA

Medicinal seeds were finely ground using a spice grinder until they were fine enough to pass through a sieve (250 µm). About 5 g of this seed power was extracted and analyzed with the same methods described above.

### 3.6. Statistical Analysis

The software SPSS (SPSS version 15.0; SPSS Inc., Chicago, IL, USA, 2006) was used for data analysis. Comparisons of the mean values of data were performed using a Fisher’s Least Significant Difference (LSD) test with a significance level at α = 0.05.

## 4. Conclusions

Despite the importance of medicinal seeds globally, little research has been done to characterize the mycobiota and occurrence of mycotoxigenic fungi in these seeds. The results presented here provide, for the first time, information about the presence and distribution of mycotoxins (aflatoxins and OTA) in commonly used medicinal seeds as well as mycotoxigenic fungi and their ability to produce these mycotoxins. For surface-sterilized medicinal seeds, *C. globosporum*, *M. trigonosporus* and *A. alternata* were most commonly detected. Patterns indicative of co-infection with *C. globosporum* and *Penicillium* spp., *C. globosporum* and *Eurotium* spp., *Eurotium* spp. and *Aspergillus* spp., *A. niger* and *A. flavus* were observed in tangerine, lychee, and platycladi seeds. Large titers of superficial fungi were observed and low similarity was found between superficial and internal mycobiota. UPLC-MS/MS analysis indicated that the occurrence of AFB1, AFB2, AFG1, AFG2, and OTA in commonly used medicinal seeds was rare, but when they were detected, they were detected at high levels. AFB1 was detected in platycladi seeds at 52.0 µg/kg, and OTA was only detected in tangerine seeds at 92.3 µg/kg. Subsequent analysis indicates that *A. flavus* and *P. polonicum* were the potential contributors to the high levels of AFB1 and OTA contamination in platycladi and tangerine seeds, respectively. Recently, the maximum permitted levels of AFs in several medicinal herbs have been set in China. Considering the toxicological effects of OTA, a maximum permitted level in several medicinal herbs is needed.

## Supplementary Materials

Supplementary materials can be accessed at: http://www.mdpi.com/2072-6651/7/10/3858/s1.
