# Supplementary material for: Mycobiota and Mycotoxins in Traditional Medicinal Seeds from China
Source: Toxins (Basel). 2015 Sep 24;7(10):3858–75. doi: 10.3390/toxins7103858 (PMC4626707; doi:10.3390/toxins7103858)
Supplement: Supplementary file 1 [file toxins-07-03858-s001.pdf]

# Supplementary Information

**Table S1.** *Aspergillus flavus* strain Platycladi-3-3 partial beta-tubulin gene sequence [a].

| Sequence |
|---|
| 1 CTCGGATGTG TCCTGTTATA TCTGCCACAT GTTTGCTAAC AACTTTGCAG GCAAACCATC |
| 61 TCTGGCGAGC ACGGCCTTGA CGGCTCCGGT GTGTAAGTAC AGCCTGTATA CACCTCGAAC |
| 121 GAACGACGAC CATATGGCAT TAGAAGTTGG AATGGATCTG ACGGCAAGGA TAGTTACAAT |
| 181 GGCTCCTCCG ATCTCCAGCT GGAGCGTATG AACGTCTACT TCAACGAGGT GCGTACCTCA |
| 241 AAATTTCAGC ATCTATGAAA ACGCTTTGCA ACTCCTGACC GCTTCTCCAG GCCAGCGGAA |
| 301 ACAAGTATGT CCCTCGTGCC GTCCTCGTTG ATCTTGAGCC TGGTACCATG GACGCCGTCC |
| 361 GTGCCGGTCC CTTCGGTCAG CTCTTCCGTC CCGACA |

[a] The 396-bp fragments of partial beta-tubulin gene were sequenced from AFB1 and AFB2 produced fungus, *Aspergillus flavus* strain Platycladi-3-3. This sequence was deposited in GenBank with accession number KT737451. The strain was deposited in Institute of Medicinal Plant Development, Chinese Academy of Medical Sciences, Beijing, China.

**Table S2.** *Penicillium polonicum* strain Tangerine-3-3 partial beta-tubulin gene sequence [a].

| Sequence |
|---|
| 1 ATTGACAGGT TACTAACTCG ATTACAGGCA AACCATCTCT GGCGAGCACG GTCTCGATGG |
| 61 CGATGGACAG TAAGTTTTAA TGGTGATGTG GGTTTCCGGT AGATCACACG TCTGATATCT |
| 121 TGCTAGGTAC AATGGTACCT CCGACCTCCA GCTCGAGCGT ATGAACGTCT ACTTCAACCA |
| 181 TGTGAGTCCA ATCACTGGAA ACCGAATAAT CGTGCATCAT CTGATCAGAT GTTTTTCTTT |
| 241 GATATCTAGG CCAGCGGTGA CAAGTACGTT CCCCGTGCCG TTCTCGTCGA TTTGGAGCCT |
| 301 GGTACCATGG ACGCTGTCCG CTCCGGTCCT TTCGGCAAGC TTTTCCGCCC CGACAACTTC |
| 361 GTCTTCGGTC AGTCCGGTGC TGGTAACAAC TGGGCCAAGG GTCAC |

[a] The 405-bp fragments of partial beta-tubulin gene were sequenced from OTA produced fungus, *Penicillium polonicum* strain Tangerine-3-3. This sequence was deposited in GenBank with accession number KT737453. The strain was deposited in Institute of Medicinal Plant Development, Chinese Academy of Medical Sciences, Beijing, China.

**Table S3.** *Penicillium polonicum* strain Lychee-2-2 partial beta-tubulin gene sequence [a].

| Sequence |
|---|
| 1 TACTAACTCG ATTACAGGCA AACAATCTCT GGCGAGCACG GTCTCGATGG CGATGGACAG |
| 61 TAAGTTTTAA TGGTGATGGG GGTTTCCGGT AGATCACACG TCTGATATCT TGCTAGGTAC |
| 121 AATGGTACCT CCGACCTCCA GCTCGAGCGT ATGAACGTCT ACTTCAACCA TGTGAGTCCA |
| 181 ACGACTGGAA ACGAATAATC GTGCATCATC TGATCAAATG TTTTTCTTTG ATAATCTAGG |
| 241 CCAGCGGTGA CAAGTACGTT CCCCGTGCCG TTCTCGTCGA TTTGGAGCCC GGTACCATGG |
| 301 ACGCTGTCCG CTCCGGTCCT TTCGGCAAGC TTTTCCGCCC CGACAACTTC GTCTTCGGTC |
| 361 AGTCCGGTGC TGGTAACAAC TGGGCCAAG |

[a] The 389-bp fragments of partial beta-tubulin gene were sequenced from OTA produced fungus, *Penicillium polonicum* strain Lychee-2-2. This sequence was deposited in GenBank with accession number KT737452. The strain was deposited in Institute of Medicinal Plant Development, Chinese Academy of Medical Sciences, Beijing, China.
